# Promoting contraceptive use among unmarried female migrants in one factory in Shanghai: a pilot workplace intervention

**DOI:** 10.1186/1472-6963-7-77

**Published:** 2007-05-31

**Authors:** Xu Qian, Helen Smith, Wenyuan Huang, Jie Zhang, Ying Huang, Paul Garner

**Affiliations:** 1 Effective Health Care Research Programme, Department of Maternal and Child Health, Fudan University School of Public Health, 138 Yi Xue Yuan Road, Shanghai 200032, P.R. China; 2 International Health Group, Liverpool School of Tropical Medicine, Pembroke Place, Liverpool, L3 5QA, UK

## Abstract

**Background:**

In urban China, more single women are becoming pregnant and resorting to induced abortion, despite the wide availability of temporary methods of contraception. We developed and piloted a workplace-based intervention to promote contraceptive use in unmarried female migrants working in privately owned factories.

**Methods:**

Quasi-experimental design. In consultation with clients, we developed a workplace based intervention to promote contraception use in unmarried female migrants in a privately owned factory. We then implemented this in one factory, using a controlled before-and-after design. The intervention included lectures, bespoke information leaflets, and support to the factory doctors in providing a contraceptive service.

**Results:**

598 women participated: most were under 25, migrants to the city, with high school education. Twenty percent were lost when staff were made redundant, and implementation was logistically complicated. All women attended the initial lecture, and just over half the second lecture. Most reported reading the educational material provided (73%), but very few women reported using the free family planning services offered at the factory clinic (5%) or the Family Planning Institute (3%). At baseline, 90% (N = 539) stated that contraceptives were required if having sex before marriage; of those reporting sex in the last three months, the majority reporting using contraceptives (78%, 62/79) but condom use was low (44%, 35/79).

Qualitative data showed that the reading material seemed to be popular and young women expressed a need for more specific reproductive health information, particularly on HIV/AIDS. Women wanted services with some privacy and anonymity, and views on the factory service were mixed.

**Conclusion:**

Implementing a complex intervention with a hard to reach population through a factory in China, using a quasi-experimental design, is not easy. Further research should focus on the specific needs and service preferences of this population and these should be considered in any policy reform so that contraceptive use may be encouraged among young urban migrant workers.

## Background

Pregnancy in single women is increasing in China, and in urban areas, particularly among migrants, abortion appears common, and women may be using it as a method of contraception [1]. A systematic review of surveys from five provinces in China showed over 54% of urban women interviewed prior to marriage had experienced sexual intercourse; 12%-32% had been pregnant, and almost all of these had an induced abortion [2]. In Beijing, pregnancy in unmarried women accounts for about 40% of total abortions [3] and it is reported over 70% of unwanted pregnancies are caused by unprotected sex, while others report failure of contraceptive methods [4].

Methods of temporary contraception are widely available in urban China; condoms are available at supermarkets and in factory clinics; contraceptive pills are sold in pharmacies and government family planning services. The cost of contraceptives varies; third generation contraceptives (currently the most popular in Shanghai) are the most expensive and probably unaffordable for unmarried young people. In state owned factories, doctors provide contraceptive services with education, counselling and contraceptive provision. However, in privately owned factories, doctors are employed to provide basic first aid rather than health promotion activities, so contraceptive services are more limited.

Yet it is precisely in these private factories where the need is likely to be highest: changes towards a market oriented economic policy means there are far more privately owned factories, and they often employ the migrant or 'floating' population which comprises young women who are highly mobile, hard to reach, and are isolated from comprehensive family planning services.

It is in these populations that research indicates that the younger age groups lack knowledge and awareness of effective contraception methods and perceive induced abortion is a method of contraception [5]. Results from a survey in Shanghai suggest that women undergoing induced abortion were often not aware of emergency contraception [6]. In urban migrant populations in Shanghai, about half of unmarried young women have experienced pregnancy, and 40% of them chose not to attend legal clinics for safe abortion [7]. Other research indicates incomplete knowledge of reproductive health issues and contraceptive methods, especially among younger people, and among those with low levels of education and income [8,9]. The National Family Planning Program's current policy for contraception targets married couples; contraceptive counselling and services for young, unmarried people in urban China are limited.

We decided to pilot a workplace-based intervention to promote contraceptive use in unmarried female migrants working in privately owned factories. We measured whether women used the intervention and how acceptable it was; we also explored women's responses to questions about their knowledge and behaviour at baseline and follow-up.

## Methods

Our main objective was to evaluate the feasibility of this intervention rather than impact on knowledge and behaviour. We developed a workplace based intervention to promote contraceptive use in unmarried female migrants in consultation with women working in a privately owned factory. We then implemented this in the factory, using a quasi experimental design. We used surveys before and after the intervention, and semi-structured interviews and focus group discussions to evaluate feasibility and acceptability.

### Study site

One privately-owned factory in a suburban district in Shanghai was selected for the intervention; the district Centre for Disease Control (CDC) helped to locate a factory which employed mainly young migrant workers, and where the managers were willing to cooperate with the study. The factory is Taiwanese owned, employs 5000 people, assembles mobile phones, and employs women exclusively in the workshop. We selected the biggest department in the main campus of the factory as the intervention department. The control group was two other departments (in order to maximise the sample size): one was the only department outside of the main campus but with limited workers (only 131 study participants) and the other (with 127 study participants) was in the main campus but located in a building separate from the intervention group. Because of the way the factory was organised in shifts, the workers from different departments rarely communicated with each other. The factory has one clinic, on the main campus, with two doctors who provide first aid, occupational health services, and a limited family planning service (free distribution of condoms only). A doctor visits departments outside the main campus once a week to provide the same services.

### Sample size

We used the contraceptive prevalence rate from a study of unmarried youth in Shanghai [10] to calculate the sample size. For the purpose of this pilot study, we estimated an increase in contraceptive use every time from 20–40% after the intervention, and considered a 10% loss with 95% confidence and 90% power. The total sample size for each group was calculated at 245. The actual sample recruited was 340 unmarried women in the intervention group and 258 in the control group.

### Intervention

The intervention was designed to promote use of effective contraceptives among unmarried female migrant factory workers (aged 16–30 years). We consulted with providers and clients within the study factory in designing the intervention. For the providers, we interviewed the factory manager and two factory doctors at baseline to understand the health situation of young female factory workers and the feasibility of implementing the intervention. For the clients, we used qualitative methods (3 focus groups and 4 individual interviews) in another department not taking part in the study. The results indicated that: this group of women usually acquire reproductive health knowledge from magazines and the radio; those using contraception mainly used natural family planning methods and condoms; they demanded more information on reproductive physiology, contraceptive methods and STD prevention. Based on these findings, we designed the intervention to include information on the range family planning methods available and reproductive physiology, and delivered it in ways the young women suggested. The intervention period was designed for six months; baseline data were collected during July-August 2004 and we followed up in January-February 2005. The intervention had five components: 

**1. Training factory doctors in family planning service delivery:** We assumed  the two existing factory doctors were the best able to spread knowledge and  information about family planning, and offer counselling as well as contraceptive  services to unmarried women working at the factory. At the intervention site, we  arranged for one doctor to receive professional training in lecturing on  contraceptive techniques; the other doctor was trained in contraceptive use and  counselling for young people at Shanghai Institute of Family Planning Technical  Instruction (SIFPTI).  

**2. Lectures given by experts:** We invited experts from Shanghai Institute of  Family Planning Technical Instruction to deliver two sets of lectures, one about  reproductive physiology and barrier methods, the other about oral and  emergency contraceptive and sexually transmitted infection prevention. Both sets  were repeated several times to ensure coverage of all staff across shifts.  

**3. Disseminating educational materials:** The research team from Fudan  University School of Pubic Health, in consultation with experts from the Shanghai  Institute of Family Planning Technical Instruction, designed a booklet especially  for unmarried female youth named “safe sex- guidebook for female youth” (see  figure 1). The booklet was piloted in focus group discussions with women from  another department not taking part in the study; some text and figures relating to  the menstrual cycle and condom use were modified to improve understanding  among the target population. The contents of the booklet complemented the  lectures. The booklets were only disseminated to the intervention group after the  second set of lectures. If the subjects did not participate in the second set of  lectures, they received booklets from the workshop manager.  

**4. Knowledge quiz with prize:** A knowledge quiz, based on the reading material  content, was held during the late intervention period. A test paper together with instruction were put in envelopes and sent out by the workshop manager to  participants in the intervention group. Participation was voluntary and the young  women were asked to return their test paper in a specially provided mailbox in  the clinic within ten days. First, second and third prizes were awarded to those  achieving the highest scores.  

**5. Free contraceptives and a counselling service **were provided as part of the  intervention; condoms were donated by the district Family Planning Commission,  contraceptive pills were provided by the research project, and counselling was  available with doctors at the factory clinic and Shanghai Institute of Family  Planning Technical Instruction. Condoms were distributed after each lecture, and  free contraceptive pills and emergency contraceptives were obtained using a free  service card at the Institute (via an agreed contract) and the factory clinic. The  free service card contained the opening hours, address of the Institute, and an  existing free counselling hotline number (run by the Institute).  

Women working in the control department did not receive any component of the  intervention, and continued to receive the regular service provided by the factory  clinic.

### Data collection

#### Questionnaire

At baseline and at follow up at six months, we asked all unmarried women to anonymously complete a questionnaire to determine reproductive health-related knowledge, frequency of sexual intercourse, and contraceptive use before and after the intervention period. The same questionnaire was completed by participants in the intervention and control departments. In the intervention group, questionnaires were distributed prior to lectures provided as part of the intervention; in the control group we supervised self-completion at baseline, and distributed questionnaires at follow-up for self-completion and return to a central location. Information about the study and the consent form were provided on the front of the questionnaire, and participation in the study was voluntary. We asked women to state their marital status on the front of the questionnaire and we excluded married women from the study. At baseline, we disseminated 611 questionnaires and received 598 completed questionnaires (98% response rate); we do not have information about the reasons for non-participation. At follow up, all available participants in the intervention and group and 94% in the control group completed the questionnaire. However, changes in factory policy resulted in redundancy and large loss to follow up in the intervention (24.4% loss) and control (35% loss) groups.

#### Qualitative data

We conducted semi-structured interviews with young women in the intervention department to explore their views about the intervention, and their opinion on services for unmarried youth. The department director helped to identify young women; five for individual interview and 6–8 women in each of three group discussions. For the interviews, young women were purposefully selected to be able to give detailed information about young women's use of contraceptive services; participants with fixed boyfriends (long term, stable relationship) were invited to participate as they were more likely to be using contraceptives and have used these services. Two women refused to be interviewed; both did not want to talk openly about themselves. For the focus group discussions, we were constrained by the demands of the factory production line and had to recruit women available on the day we conducted the discussions. In focus groups we asked participants what they knew about the intervention, what they thought about it, their feedback on the materials, and their views about changes in attitude among their peer group.

All interviews and group discussions were conducted by teachers and graduate students from Fudan University School of Public Health and written informed consent was obtained. Interviews took place in the factory in a private meeting room and were tape recorded.

### Data management and analysis

We set up the quantitative database using Epidata2.1. After checking and coding questionnaires, data were entered by two students separately and crosschecked again to assure the accuracy of data. The fixed database was analyzed using SPSS11.0. Both one-way analysis and multiple-way analysis were used. One-way analysis contained t test, chi square test, nonparametric test (α = 0.05).

Interview tape recordings were initially transcribed in Chinese characters and analysed using the Framework approach [11]. Complete transcripts were translated into English; QX and HWY read through the Chinese versions and HS read the English transcripts to develop the coding index. Some of the transcripts contained very brief responses to interviewer's questions and leading questions to prompt responses. This is probably because young women had to be encouraged to participate, and only few actually spoke out about their experiences. We decided to use only data derived from non-leading questions in the analysis. We discussed possible codes and came to a consensus on the coding framework to be used in the analysis; the coding framework was also informed by topics covered in interview guidelines. We used MaxQDA software to code the transcripts and search and retrieve data. We charted coded responses in a matrix to look across the data, and grouped coded segments into categories. We refined categories into main themes and discussed the dimensions of each theme together as a group. We provide a descriptive account of each theme in this paper using illustrative quotes; all references to individuals by name have been changed to protect their identity.

### Ethical approval

The study was approved by the Institute Review Board in the School of Public Health at Fudan University which is registered with the office for human research protections and has a federal wide assurance (approval no. IRB#04-08-0018).

## Results

Our main objective was to evaluate the feasibility of this intervention, and so we report here on a) practical problems encountered in implementation; b) baseline survey results; c) uptake of the intervention; d) trends in self-reported knowledge and behaviour; e) acceptability of the intervention among the target population.

### a) Practical problems encountered in implementation

The implementation was not straightforward, and we ran into the following specific problems:

i) The migrant or 'floating' population working in factories means that young people move jobs frequently. In addition, during the intervention period, demand for the telephones dropped, management issued a new human resource policy, and many young women were made redundant. We did request the factory manager retain as many staff as possible in both departments during the study. The manager only agreed to keep women on in the intervention department for the duration of the study; the control group followed the factory policy and more women were fired than the intervention group during the final survey. In the intervention group we were able to track only 257/340 participants during the final survey (24% loss). According to the factory manager, most of these women left the factory for employment elsewhere (77) or to be married (6). In the control group, 168/258 participants were available for the final survey (35% loss); the main reason was the new factory policy and reduced workforce. The loss to follow up resulted in a small sample size with insufficient power to analyze the quantitative data for some indicators.

ii) At baseline, the questionnaire survey was completed by participants in an open room prior to the lecture; about 2/3 of the participants completed the final survey in these conditions – the others received the questionnaire in an envelope to take away to complete and return the next day. This is because the women were needed on the assembly line and the department leader refused permission for them to attend. Self-completion of the questionnaire outside of the factory by this group of women might affect the follow-up results, since participants could openly consult friends and colleagues and the provided reading materials.

iii) The factory doctor who was trained in counselling techniques left the factory (she was made redundant) during the last 2 months of the intervention study. This may have affected our study participant's ability to use the service in the factory clinic.

iv) For the first lecture, the human resource manager organized the meeting and encouraged young women to attend. For the second set of lectures, attendance was voluntary and invitations were sent out to individuals. We designed it this way to test the demand for knowledge and constraints to delivering the intervention within the factory; but this had important implications for attendance at the second lecture and meant we had to distribute follow-up questionnaires in envelopes.

### b) Baseline survey results

Table 1 shows the characteristics of the intervention and control groups at baseline. Women were young, most were recent migrants, and had a high school education; education to high school level is typical of migrant workers because this is a usual requirement for employment in factories in urban areas. The two groups were essentially similar, except there were more women under 20 years in the intervention group.

**Table 1:** Characteristics of survey sample at baseline

	**Intervention** **N = 340** **(%)**	**Control** **N = 258** **(%)**	**P value**
**Age**			0.046
<20 years	32.0	22.9	
20–24 years	61.5	69.4	
>24 years	6.5	7.7	
**Lived in Shanghai**			0.014
<6 month	5.0	0.8	
6 month – 2 year	69.7	73.6	
>2 year	25.3	25.6	
**Education**			0.776
<10 grade	6.8	8.2	
High school	90.9	89.9	
College and above	2.3	1.9	

**Has a boyfriend**	55.2	51.6	0.402

Attitudes towards pre-marital sex-related behaviour were similar in the intervention and control groups (table 2). Nearly 60% of the women disapproved of premarital sex; about 80% of them disapproved of premarital pregnancy and approximately 90% believed contraceptive use for premarital sex is required.

**Table 2:** Reported attitudes to premarital sex-related behaviour at baseline and follow-up

	**Baseline**			**Follow-up**		
	
	**Intervention** **N = 340**	**Control** **N = 258**	**P**	**Intervention** **N = 257**	**Control** **N = 168**	**P**
	**(%)**	**(%)**		**(%)**	**(%)**	
**What do you think about a colleague having sex before marriage?**			0.848			0.509
Fine by me	13.8	13.2		12.5	6.4	
Don't care	27.4	29.5		28.0	31.0	
Disapprove	58.8	57.3		59.5	62.6	
**What do you think about a colleague who becomes pregnant before marriage?**			0.323			0.793
Fine by me	4.5	4.7		4.7	5.2	
Don't care	12.1	16.3		14.0	14.7	
Disapprove	83.4	79.0		81.3	80.1	
**Should people use contraceptives if having sex before marriage?**			0.202			0.913
Not necessary	5.0	3.1		3.9	3.6	
Don't care	6.8	4.3		5.1	7.5	
Necessary	88.2	93.6		91.0	88.9	

A total of 79 women reported having sex within the past three months. The majority had used some form of contraception (78%; 62/79) but condom use was low (44%; 35/79).

### c) Uptake of the intervention

Through the questionnaire we collated data on uptake of the various components of the intervention (table 3). The two sets of lectures were held five times; attendance at the first lecture (reproductive physiology and barrier methods) was 100%, but just over half of young women in the intervention group reported attending the second lecture on oral and emergency contraceptive and sexually transmitted infection prevention. Each participant received a booklet on safe-sex; 73% reported reading the booklet; and 195 (54%) women in the intervention group reported taking part in the voluntary knowledge quiz. Condoms were distributed after each lecture; 334 packages of condoms (each containing one dozen condoms) were taken by the participants. Very few women reported using the free family planning services (oral contraceptive pills, emergency contraception and counselling) offered at the factory clinic (5%) and the Family Planning Institute (3%).

**Table 3:** Self-reported participation in the intervention

**Intervention components**	**No. of subjects (%)**
Attended two sessions of lectures	140/257 (54)
Read the booklet	187/257 (73)
Found the booklet very useful	124/187 (66)
Kept the free service card	181/257 (70)
Used the free service in the factory clinic	14/257 (5)
Used the free service at the Shanghai Institute of Family Planning Technical Instruction	9/257 (3)

### d) Trends in knowledge and behaviour

Attitudes towards pre-marital sex-related behaviour did not change significantly in either group at follow-up (see table 2). We examined women's knowledge in the questionnaire using 18 questions relating to reproductive physiology, contraception, induced abortion, and STD/AIDS; we used these questions to determine a 'knowledge score' for each participant. The maximum score obtainable was 100 (based on correct answers to all 18 questions). The median score in the control group did not change, but the knowledge score in the intervention group increased significantly from 17.50 at baseline to 38.13 at follow-up (p = 0.000).

There was no difference between the intervention and control groups in terms of sexual behaviour. The number of women in both groups reporting having sex within the last 3 months did not change between baseline and follow-up (table 4). In women who had ever had sex in the last 3 months, contraceptive use increased from 70% to 93% within the intervention group, and condom use in the last three months increased significantly from 41% at baseline to 70% at follow-up in intervention group.

**Table 4:** Change in sexual behaviour in the last three months

**Sex related behaviour**	**Baseline**			**Follow-up**		
	
	**Intervention** **(%)**	**Control** **(%)**	**P**	**Intervention** **(%)**	**Control** **(%)**	**P**
Have had sex with 3 months	44/311 (14)*	35/231 (15)**	0.743	27/239 (11)^†^	15/159 (9) ^‡^	0.553
Have used any contraception in the last three months^#^	31/44 (70)	31/35 (89)	0.052	25/27 (93)^⊕^	13/15 (87)	0.531
Have used condoms in the last 3 months^#^	18/44 (41)	17/35 (49)	0.496	19/27 (70)^⊕^	6/15 (40)	0.055

*29 non-response; **27 non-response; † 18 non-response; ‡ 9 non-response;

# denominator is the number of women who have ever had sex within 3 months.

⊕ intervention group, baseline compared to follow up, chi square test, p < 0.05.

### e) Acceptability of the intervention

We explored the acceptability of the intervention using semi-structured interviews and focus group discussions with young female factory workers in the intervention group. We asked specifically for their feedback on the intervention and elicited their opinions on services for unmarried young people; three main themes emerged from the analysis.

#### Theme 1. Privacy and anonymity are important to young women

When discussing the components of the intervention and the contraceptive services provided, women frequently used the phrase '*bu hao yi si' *(meaning to be embarrassed or feel uncomfortable). This was mentioned in relation to using the free family planning service at the factory clinic and the Family Planning Institute, for example, "I think probably because it is a free service, some people felt uncomfortable [bu hao yi si] about taking the service without paying". Others mentioned feeling embarrassed about using the factory clinic because it was situated in their working environment, for example, "I am working here, so I am definitely shy [bu hao yi si] to go to the factory clinic". Another woman described how the factory clinic was inappropriate for providing the service because individual privacy is compromised and doctors may judge (in Chinese *'yan guang'*) young peoples behaviour: "...some young girls don't want people know about her boyfriend; if they live together, she will not go to the factory clinic to take condom. The best place is where no-one can recognize me, so I will go to drug store. I also think if the young girl in our factory has emergency contraception need, she will not call the hotline. She will find the closest drug store to secretly get medicine, because she is a migrant. She doesn't know if Dr A will have good attitude or not. I feel she will think like this. If I was that age, I am afraid of being judged by others [yan guang]. I remember once I got gastroenteritis to have vomiting, the hospital doctor's judgement [yan guang] was that I was pregnant because she asked me if my menstruation is normal."

Women also referred to embarrassment or '*bu hao yi si' *when describing their views about special services for unmarried youth. There were mixed opinions about services for unmarried youth, but many respondents emphasised the importance of feeling comfortable with the service provider and maintaining privacy for young people:

"I think the service is necessary. Now many things of this kind happen... So I think the service is more important. At the beginning, if we didn't have the knowledge training, we would feel uncomfortable [bu hao yi si] to use the service. If I didn't listen to your lecture but someone told me about counselling, the hotline, or a place for the service, I may not call or go. But if we learned from and communicated with you, and we trusted you, then I may go to find you. If a girl has a gynaecological problem, she will not feel comfortable [bu hao yi si]. If there is a service only for unmarried youth and doctors in it are friendly, that is good, and I think it's necessary". (Interview respondent)

"I don't think it is good. Though some people maybe don't care, but if unmarried youth go to this kind of clinic, people without open minds will have other thoughts about the youth." (Focus group discussion participant)

*"I think the clinic you just said is very important. Now the youth mature earlier. Adolescents easily get excited, and then experiment. But they are not willing to tell their parents. So the clinic should be set up. But the door of the clinic should not state 'for unmarried youth', or have a clear advert, it should protect privacy for the user." (Focus group discussion participant)*.

#### Theme 2. Constraints to using services provided in the intervention

Women we interviewed mentioned that the factory shift-work system prevented them attending the lectures, particularly if a lecture was timetabled during their leisure time. A typical comment was, "generally we will not come to the factory if we are not on duty, even if the lecture is arranged. If the lecture is during work time, and we are not busy, we will come" (focus group discussion participant). Others said they did not attend because they are busy with work, "I am busy in my work and don't have time to go down to the lecture". Other women said they did not receive any notice about the second lecture, so did not attend.

Some women told us they had no need to use the services. For example, when asked if they had used the free service card, women in one focus group explained they "did not meet any problems" or, "I have no need". Women who were interviewed expressed similar views, one said she hadn't had any need to use the card but went on to explain that she will use different services for different problem: "if I get pregnant, I will go to see Dr A [at SIFPTI], if it is for a small problem such as gynaecological infection. I will consult Dr B [factory doctor] if I only want to take some condoms or pills, such kind of small things I will not go to see Dr A". Another woman explained, "I didn't meet any problems so I didn't think about using this card, if I have real trouble I definitely will call [the hotline number on the service card]". A minority of women in one focus group and one interview said the Institute-based service was located too far away, for example, "we have to take a bus to go to the Institute [SIFPTI], it is inconvenient except for emergency contraceptives". One woman said she doubted whether the free products were genuine, so she hadn't used the service: "...some may not be willing to take [the free contraceptives], because you wonder if the free contraceptives are good or will have side effects. This definitely is an influencing factor".

#### Theme 3. Demand for specific reproductive health knowledge

Interview and group discussions suggested that women had learnt from the lecture and read the booklet (they freely recalled the lecture content and commented on the booklet), but the dialogue suggested a demand for more specific information and knowledge.

Several women made positive remarks about the lectures and reading material and indicated their knowledge about contraception had increased:

"I learned how to protect myself after your lecture, I know how to use condom now." (Focus group discussion)

"I felt I knew about condom and contraceptives before. But I don't know the effect of this contraception, or that there are long term and short term contraceptive pill and even have emergency contraceptive pill. Before I only know two types of contraception but don't know the detail about them. Now I am clear and can choose it by myself. If I want to buy them, I can say clearly which kind I want." (Interview)

"The contents are fine. I don't know all this before the lectures. I even can't recognize condoms before." (Focus group discussion)

Others suggested that the intervention had been beneficial and indicated they were not just 'curious' but 'wanted to know' about reproductive health issues:

"I want to know about this. So I am willing to read this booklet." (Focus group discussion)

"I want to know more about this; it is not bad for me." (Focus group discussion)

"I think it is necessary to have health education in orientation training. It is definitely not a waste of time to listen your lecture. It definitely has benefit" (Interview)

"To read this booklet is not because I am curious, but I want knowledge about this aspect." (Focus group discussion)

"To learn about these things has no harm. I feel it was necessary for me to listen to the lectures. I read the booklet in the evening the day I got it. I also gave it to my boyfriend to read. We don't talk too much, so I let him read it." (Interview)

There appeared to be demand for the material content to have 'more breadth and depth':

"I feel this booklet doesn't have enough detail, it is just a simple introduction. For example, it only introduces several infectious diseases and gives the concept of prevention. It doesn't give the details." (Focus group discussion)

"I think it is better for us to have more knowledge on gynaecological problems such as common female physiological problems, common diseases, how to prevent them and how to self-care in daily life." (Focus group discussion)

"The booklet is just a brief introduction, it doesn't have detailed contents. I want to know more about gynaecological problems, especially how to prevent them." (Interview)

Others specifically mentioned they would like more information about HIV/AIDS:

"I feel this booklet generally is quite good. But targeted at unmarried females, I think it should include more contents about safe sex. You have some detail contents on AIDS and STDs, but personally I feel it leaves me wanting to know more." (Focus group discussion)

"I want to know about AIDS, for example, how people get AIDS and what we should do for prevention in daily life." (Interview)

"I want the contents to be broader and more detailed, such as AIDS, why people get AIDS, what we should do for the prevention in daily life. In terms of AIDS, I only heard about it but don't know much about it." (Focus group discussion)

## Discussion

We have not identified any attempt at a quasi-experimental design to deliver a reproductive health intervention targeted at a hard-to-reach population in the private sector in China. Our experience suggested that they are not easy to conduct. In particular, the large loss to follow up due to factory redundancies resulted in a smaller sample size with insufficient power (fewer than the required 245 participants in each group) to detect a difference in contraceptive use. It is therefore inappropriate to make inferences about the effect of the intervention on behaviour and knowledge. For example, the increase in contraceptive use in the intervention group may be an over estimate, and no change in the control group could be due to insufficient power in the study design. Although those participants lost to follow up are likely to be similar in age and education level to those analysed, we are unable to compare their characteristics because questionnaires were completed anonymously. We did not estimate cost, or examine factory managers' willingness to support workplace family planning services in this pilot study.

### Interpretation

The data presented here questions the assumption from previous research in China that young people lack knowledge and awareness of effective contraception methods. Our baseline data on attitudes indicated a perceived need for contraceptive use in unmarried youth; around 90% of women in both groups said contraceptive use was necessary in premarital sex. This indicates factors other than knowledge might be important in determining use of contraceptives in this population. The qualitative findings indicate that 'shyness' or embarrassment were important reasons why these female factory workers did not use the services provided as part of the intervention. Feeling comfortable with service providers and maintaining privacy were also regarded as important features of services for unmarried youth. These factors, rather than lack of knowledge, appear to represent considerable barriers to use of contraceptives by young unmarried women.

Implementation of workplace based interventions in China is not easy given the rapid changes in economic policy and sudden changes in human resource demand at factory level. The involvement and cooperation of the factory leader is required for successful implementation; it is important they to agree to the principles of the intervention and support its implementation. Cooperation of factory leaders in organising shift times for the workers involved can facilitate data collection. We learnt that changes to factory employment policies during the lifetime of an intervention study are largely unpredictable and can result in significant loss to follow-up.

Our intervention involved training factory doctors in family planning counselling for young unmarried women; this is a task outside their usual remit at privately owned factories (occupational health and family planning for married couples). These doctors know the young workers well, but it is unclear from our findings whether this is the right way to deliver family planning services to young unmarried migrant workers. The survey results show very low use of the free service provided by the factory doctors and qualitative findings suggest women were embarrassed to use the services in the factory because the doctor was familiar and their privacy was compromised.

Qualitative data indicate young women seek help based on their current needs and not simply because a service is provided for free; some were even hesitant about using 'free' contraceptives, questioning whether they were genuine. Women commented on the need to feel comfortable with family planning providers in an environment that protects their privacy, and that the most accessible place to obtain contraceptives would be a drug store nearby their home.

Despite not using the free services provided in the intervention, the reading material seemed to be popular and young women working at the factory expressed a need for more specific reproductive health information, particularly on HIV/AIDS. Based on the trend of increased condom use detected in this study, and evidence of increasing incidence of STI's among younger age groups in China, it is likely that condoms are the most appropriate contraceptive method to promote among the unmarried migrant population [12].

## Conclusion

Our study has demonstrated that providing contraceptive services to unmarried migrant women working in privately owned factories is not easy. Unmarried women prefer family planning services that protect their privacy, where they can be anonymous, and use service providers they trust. Further research should focus on the specific needs and service preferences of this population and these should be considered in any policy reform so that contraceptive use may be encouraged among young urban migrant workers.

## Competing interests

The author(s) declare that they have no competing interests.

## Authors' contributions

QX identified the study question, and developed the protocol with HS and PG. WH collected and analyzed data and help write the paper. JZ collected data and YH conducted part of the data analysis. All authors participated in the interpretation of data and writing the final manuscript.

## Pre-publication history

The pre-publication history for this paper can be accessed here:


